# The Effect of Niobium Doping on the Electrical Properties of 0.4(Bi_0.5_K_0.5_)TiO_3_-0.6BiFeO_3_ Lead-Free Piezoelectric Ceramics

**DOI:** 10.3390/ma8125457

**Published:** 2015-12-02

**Authors:** John G. Fisher, Seo-Hee Jang, Mi-So Park, Hengyang Sun, Su-Hyun Moon, Jong-Sook Lee, Ali Hussain

**Affiliations:** 1 School of Materials Science and Engineering, Chonnam National University, Gwangju 500-757, Korea; 8338644@naver.com (S.-H.J.); miso8652@naver.com (M.-S.P.); sun_hy0925@126.com (H.S.); suhyunmoon7@gmail.com (S.-H.M.); jongsook@jnu.ac.kr (J.-S.L.); 2 School of Advanced Materials Engineering, Changwon National University, Changwon, Gyeongnam 641-773, Korea; alihussain_phy@yahoo.com

**Keywords:** lead-free piezoelectric, BiFeO_3_, Nb, dielectric properties, piezoelectric properties

## Abstract

Ceramics in the system (Bi_0.5_K_0.5_)TiO_3_-BiFeO_3_ have good electromechanical properties and temperature stability. However, the high conductivity inherent in BiFeO_3_-based ceramics complicates measurement of the ferroelectric properties. In the present work, doping with niobium (Nb) is carried out to reduce the conductivity of (Bi_0.5_K_0.5_)TiO_3_-BiFeO_3_. Powders of composition 0.4(K_0.5_Bi_0.5_)Ti_1−*x*_Nb*_x_*O_3_-0.6BiFe_1−*x*_Nb*_x_*O_3_ (*x* = 0, 0.01 and 0.03) are prepared by the mixed oxide method and sintered at 1050 °C for 1 h. The effect of Nb doping on the structure is examined by X-ray diffraction. The microstructure is examined by scanning electron microscopy. The variation in relative permittivity with temperature is measured using an impedance analyzer. Ferroelectric properties are measured at room temperature using a Sawyer Tower circuit. Piezoelectric properties are measured using a *d*_33_ meter and a contact type displacement sensor. All the samples have high density, a rhombohedral unit cell and equiaxed, micron-sized grains. All the samples show relaxor-like behavior. Nb doping causes a reduction in conductivity by one to two orders of magnitude at 200 °C. The samples have narrow P-E loops reminiscent of a linear dielectric. The samples all possess bipolar butterfly S-E loops characteristic of a classic ferroelectric material. Nb doping causes a decrease in *d*_33_ and *S*_max_/*E*_max_.

## 1. Introduction

Solid solutions containing perovskite BiFeO_3_ have recently been attracting attention for use as lead-free piezoelectric ceramics. This is due to the high Curie temperature (~810–820 °C) and large remnant polarization (60 μC/cm^2^ for single crystals, 100 μC/cm^2^ for thin films) of BiFeO_3_ [1,2,3,4]. BiFeO_3_ is itself piezoelectrically active, with a direct piezoelectric coefficient *d*_33_ ≈ 30 pC/N and a large peak-to-peak strain of up to 0.36% measured in S *vs.* E curves at low frequency [5,6]. Non-180° domain wall movement contributes significantly to the piezoelectric properties of BiFeO_3_, manifesting in strongly nonlinear (frequency and stress amplitude-dependent) piezoelectric properties. Domain walls are also strongly pinned by charged defects, probably acceptor-oxygen vacancy defect pairs, making the material properties dependent on the sintering and poling conditions [7,8]. This sensitivity to processing conditions, along with high conductivity, high coercive fields, and difficulties in preparing single-phase powders [8,9], means that the properties of bulk ceramic BiFeO_3_ are often inferior to those of single crystal or thin film BiFeO_3_ [3,4,8].

In order to improve the electrical properties, solid solutions of BiFeO_3_ with other perovskites such as BaTiO_3_ and PbTiO_3_ have been studied [10,11,12]. The formation of solid solutions suppresses secondary phase formation and allows the creation of morphotropic phase boundaries (MPBs) between polymorphs of the perovskite phase, with a concomitant improvement in piezoelectric properties [13]. The *x*BaTiO_3_-(1−*x*)BiFeO_3_ system has an MPB at *x* = 0.33 between rhombohedral and pseudocubic phases [14]. Compositions close to the MPB have good piezoelectric properties (*d*_33_ > 100 pC/N, bipolar strain of ~0.15% at an electric field of 6 kV/mm) and good temperature stability [10,12,14,15]. Matsuo *et al.* studied the system *x*(K_0.5_Bi_0.5_)TiO_3_-(1−*x*)BiFeO_3_ (KBT-BFO) and found it to have an MPB between rhombohedral and pseudocubic phases at *x* ≈ 0.4 [16]. Compositions at the MPB have good piezoelectric properties (*S*_max_/*E*_max_ ≈ 225 pm/V, d*_33_ ≈ 130 pm/V) and good temperature stability (*k*_33_ ≈ 0.36 at 300 °C). Mozorov *et al.* also found ceramics in this system to have good inverse piezoelectric properties, making them potentially suitable for actuator applications [17,18].

BiFeO_3_-based ceramics tend to have high leakage currents, which makes measuring the ferroelectric properties and poling the ceramic difficult. Doping with Mn was found to reduce the leakage current density of BiFeO_3_ thin films under high electric fields [19]. It was also found to reduce the dc conductivity and leakage current density of BaTiO_3_-BiFeO_3_ ceramics [12,20]. Our previous work found that MnO doping reduced the dc conductivity of 0.4(K_0.5_Bi_0.5_)TiO_3_-0.6BiFeO_3_ ceramics by three orders of magnitude [21]. The rhombohedral distortion of the unit cell was also reduced. However, MnO doping degraded the polarization *vs.* electric field and the strain *vs.* electric field behavior of the ceramics. Further work should be carried out to optimize the dopant level.

Mn can be present in perovskite ceramics in Mn^2+^, Mn^3+^ or Mn^4+^ form [22], but is thought to exist mainly in Mn^2+^ or Mn^3+^ states [23]. Mn acts as an acceptor dopant substituting for Fe^3+^ or Ti^4+^ [10]. In addition to Mn, other dopants may be used to reduce the leakage current of *x*(K_0.5_Bi_0.5_)TiO_3_-(1−*x*)BiFeO_3_. Doping with Nb was found to reduce the conductivity of BiFeO_3_ and BaTiO_3_-BiFeO_3_ ceramics by six orders of magnitude [24,25]. In the present work, we have doped 0.4(K_0.5_Bi_0.5_)TiO_3_-0.6BiFeO_3_ ceramics with Nb in order to reduce their conductivity. The effect of Nb doping on the structure, microstructure, and electrical properties of 0.4(K_0.5_Bi_0.5_)TiO_3_-0.6BiFeO_3_ ceramics will be described.

## 2. Results and Discussion

**Figure 1:**
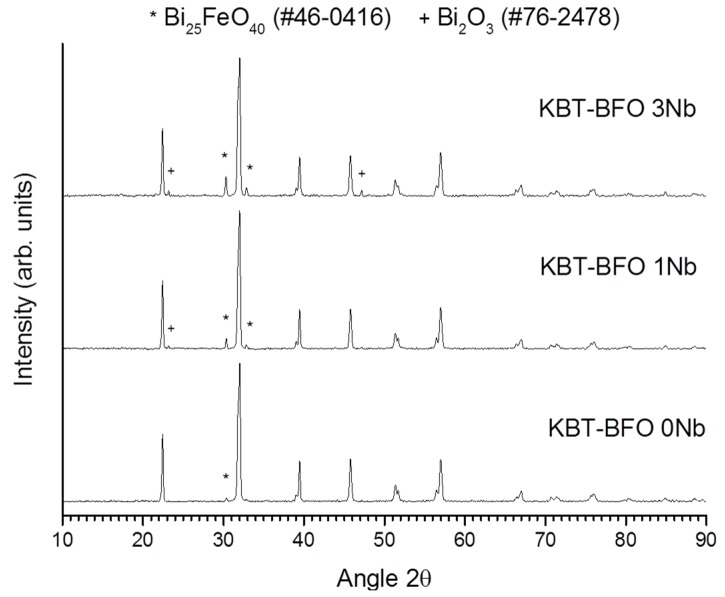
X-ray diffraction (XRD) traces of 0.4(K_0.5_Bi_0.5_)Ti_(1−*x*)_Nb_x_O_3_-0.6BiFe_(1−*x*)_Nb*_x_*O_3_ powders after calcination at 900 °C for 5 h.

**Figure 2:**
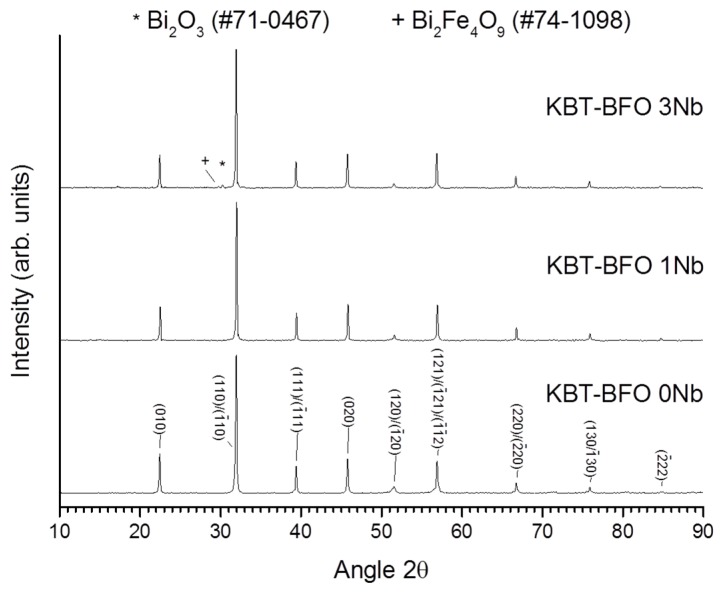
XRD traces of 0.4(K_0.5_Bi_0.5_)Ti_(1−*x*)_Nb*_x_*O_3_-0.6BiFe_(1−*x*)_Nb*_x_*O_3_ samples after sintering at 1050 °C for 1 h.

X-ray diffraction (XRD) traces of the powders after calcination at 900 °C for 5 h are shown in Figure 1. All of the patterns can be indexed with International Centre for Diffraction Data (ICDD) card #72-2321 for rhombohedral BiFeO_3_ (space group *R3m*). Second phases of Bi_25_FeO_40_ (ICDD card #46-0416) and Bi_2_O_3_ (ICDD card #76-2478) are also present. The amount of second phase increases as the amount of Nb substitution in the powders increases. XRD traces of the samples sintered at 1050 °C for 1 h are shown in Figure 2. As in Figure 1, all of the traces can be indexed with ICDD card #72-2321 for rhombohedral BiFeO_3_. The KBT-BFO 0 and 1Nb samples are single-phase, but the KBT-BFO 3Nb samples have small amounts of Bi_2_O_3_ (ICDD card #71-0467) and BiFe_2_O_4_ (ICDD card #74-1098) secondary phases. The unit cell parameters of the sintered samples are given in Table 1. The numbers in brackets are the estimated errors. Incorporation of Nb causes a small increase in the unit cell parameter and a small decrease in the rhombohedral distortion. Sample density is given in Table 1, relative to a theoretical density of 7.31 g/cm^3^ for 0.4(K_0.5_Bi_0.5_)TiO_3_-0.6BiFeO_3_ [26]. The samples all have high density and Nb addition does not have much effect on the density.

**Figure 3:**
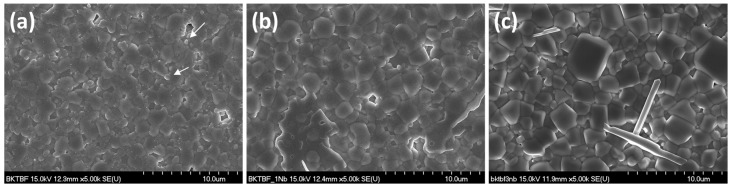
SEM micrographs of: 0.4(K_0.5_Bi_0.5_)Ti_(1−*x*)_Nb*_x_*O_3_-0.6BiFe_(1−*x*)_Nb*_x_*O_3_ samples after sintering at 1050 °C for 1 h: (**a**) *x* = 0.00; (**b**) *x* = 0.01 and (**c**) *x* = 0.03.

SEM micrographs of the samples sintered at 1050 °C for 1 h are shown in Figure 3. The microstructure of the KBT-BFO 0Nb sample consists of equiaxed grains ~1 μm in diameter (Table 1). There are fine precipitates of a second phase at the grain boundaries, as well as larger second phase grains marked by arrows in the micrograph (Figure 3a). The fine precipitates are too small to measure with energy dispersive X-ray spectrometry (EDS), but the larger second phase grains are Fe-rich compared to the matrix grains. The KBT-BFO 1Nb and KBT-BFO 3Nb samples have slightly larger grains (Figure 3b,c and Table 1). The KBT-BFO 1Nb and KBT-BFO 3Nb samples have larger second phase particles, which EDS analysis reveals to be deficient in K and Fe compared to the matrix grains.

**Table 1:** Physical properties of (K_0.5_Bi_0.5_)Ti_1−*x*_Nb*_x_*O_3_-0.6 BiFe_1−*x*_Nb*_x_*O_3_ ceramics.

Sample	Density (% Relative Density)	a Unit Cell Parameter (nm)	β (°)	Mean Grain Diameter (μm)
0.4(K_0.5_Bi_0.5_)TiO_3_-0.6BiFeO_3_	98.9 ± 0.2	0.396218 (0.0000031)	90.0195 (0.00082)	0.9 ± 0.5
0.4(K_0.5_Bi_0.5_)Ti_0.99_Nb_0.01_O_3_-0.6BiFe_0.99_Nb_0.01_O_3_	98.5 ± 1.0	0.396243 (0.0000066)	89.9882 (0.00276)	1.3 ± 0.5
0.4(K_0.5_Bi_0.5_)Ti_0.97_Nb_0.03_O_3_-0.6BiFe_0.97_Nb_0.03_O_3_	98.4 ± 0.4	0.396437 (0.0000077)	89.99 (0.00207)	1.4 ± 0.6

Secondary phases often appear in both calcined BiFeO_3_ powders and sintered BiFeO_3_ ceramics [8]. BiFeO_3_ was found to be thermodynamically unstable, decomposing to Bi_25_FeO_39_ and Bi_2_Fe_4_O_9_ upon heat treatment between 447–767 °C [27] and at 850 °C [28]. Valant *et al.* also showed that the incorporation of even a small amount of a third component that formed a solid solution with sillenite (Bi_25_FeO_39_) could induce the formation of secondary phases of Bi_2_Fe_4_O_9_ and Bi_25_FeO_39_ by shifting the composition into a three phase region [9]. The increase in the amount of secondary phases with Nb content in both the calcined KBT-BFO powders (Figure 1) and the sintered ceramics (Figure 2 and Figure 3) shows that 0.4(K_0.5_Bi_0.5_)TiO_3_-0.6BiFeO_3_ behaves in a similar manner to BiFeO_3_ regarding the effect of dopant addition on the formation of secondary phases. The secondary phases all melt at temperatures below the sintering temperature [29] and so may promote densification and grain growth by acting as a liquid phase sintering aid (Table 1).

**Figure 4:**
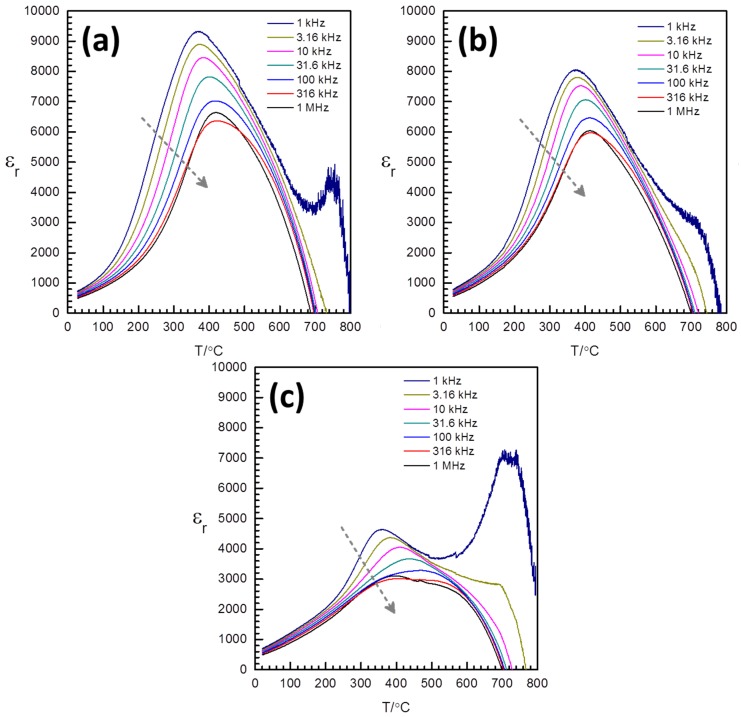
Relative permittivity *vs.* temperature for 0.4(K_0.5_Bi_0.5_)Ti_(1−*x*)_Nb*_x_*O_3_-0.6BiFe_(1−*x*)_Nb*_x_*O_3_ samples sintered at 1050 °C for 1 h: (**a**) *x* = 0.00; (**b**) *x* = 0.01; and (**c**) *x* = 0.03.

Plots of relative permittivity *vs.* temperature for the samples sintered at 1050 °C for 1 h are shown in Figure 4. The grey arrows show the direction of increasing measurement frequency. The samples show relaxor-like behavior, with the temperature of maximum relative permittivity *T_max_* increasing with the measurement frequency and the maximum value of the relative permittivity ε_rmax_ decreasing with the measurement frequency. Note that the rapid decrease in permittivity at temperatures above the peak temperature *T_max_* is, in fact, an artifact. The decrease in permittivity to negative values is determined by the lead wire inductance at high temperatures when the sample resistance is small. Further increases in permittivity at high temperature for the lower measurement frequencies (1 and 3.16 kHz) are due to dc polarization. The incorporation of Nb causes a decrease in the value of ε*_rmax_*, particularly for the KBT-BFO 3Nb sample. Nb incorporation causes an increase in *T_max_* at 10 kHz (Table 2). *T_max_* slightly increases with frequency in a relaxor-like manner, *i.e.*, *T_max_* (1 MHz) > *T_max_* (10 kHz). The ε*_r_ vs.* temperature peaks also become substantially broader for the KBT-BFO 3Nb sample, suggesting two peaks. This is indicated by the lower *T_max_* at 1 MHz than at 10 kHz in Table 2.

**Figure 5:**
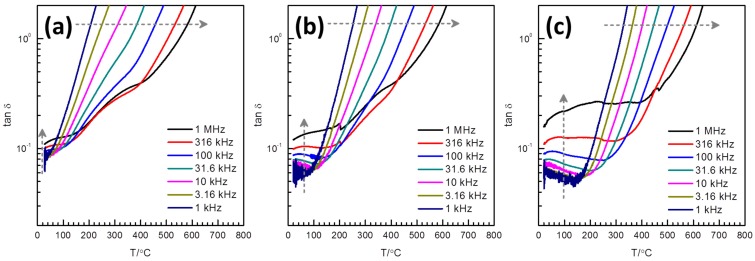
Loss tangent *vs.* temperature for 0.4(K_0.5_Bi_0.5_)Ti_(1−*x*)_Nb*_x_*O_3_-0.6BiFe_(1−*x*)_Nb*_x_*O_3_ samples sintered at 1050 °C for 1 h: (**a**) *x* = 0.00; (**b**) *x* = 0.01; and (**c**) *x* = 0.03.

Plots of loss tangent *vs.* temperature for the samples sintered at 1050 °C for 1 h are shown in Figure 5. The grey arrows show the direction of increasing measurement frequency. Note that the loss tangent is shown in a logarithmic scale. The large increase in loss tangent with temperature is due to the dc conductivity, σ_DC_. The component of loss tangent due to dc conductivity is proportional to σ_DC_/ωε. A steeper increase with temperature is indicated for the lower frequency values. The curves show a minimum point or the point of the largest slope change at low temperature, which is related to the ferroelectric transition. The temperature of this characteristic point increases with frequency and with Nb doping. Below the characteristic points, the frequency dispersion of the ferroelectric origin is indicated by the loss tangent increasing with frequency, and this dispersion becomes stronger for larger Nb doping. This is also indicated by the difference between 1 MHz and 10 kHz values of loss tangent at 40 °C in Table 2. It can be noted that the temperature range with ferroelectric dispersion in loss tangent corresponds to the temperature range with weakly dispersive relative permittivity (Figure 4). The relaxor-like dielectric response is therefore attributed to the dc leakage.

**Figure 6:**
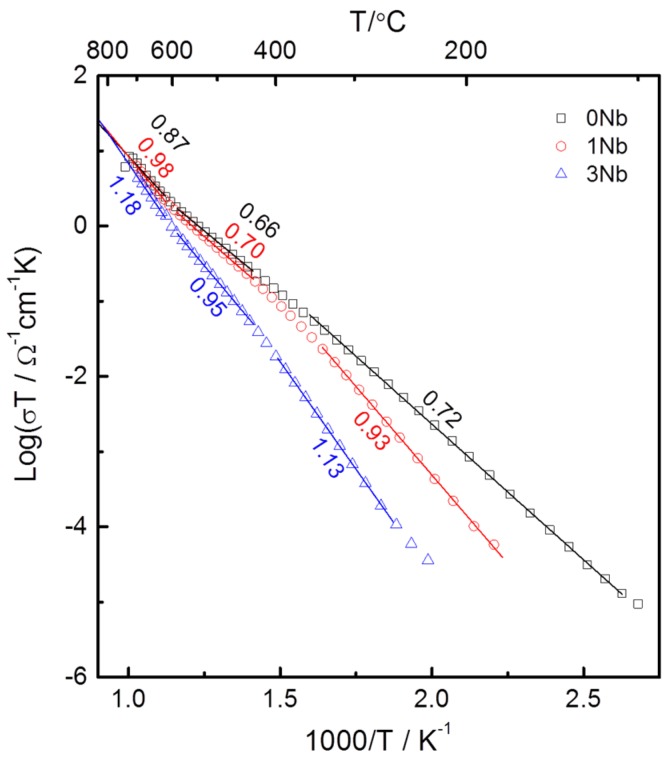
dc conductivity *vs.* 1/temperature for 0.4(K_0.5_Bi_0.5_)Ti_(1−*x*)_Nb*_x_*O_3_-0.6BiFe_(1−*x*)_Nb*_x_*O_3_ samples sintered at 1050 °C for 1 h.

The dc conductivity responsible for the large apparent loss tangent at high temperatures and apparent relaxor-like dielectric response can be obtained from the resistance magnitude of the skewed arc responses, as indicated in the previous reports [21,26]. Plots of dc conductivity *vs.* inverse temperature for the samples are shown in Figure 6. For clarity, every 20th data point is presented. The values of conductivity at 300 and 30 °C are given in Table 2. The measurements at 300 °C are directly measured or interpolated from the data. The measurements at 30 °C are extrapolated from the data. Changes in slope around 400 °C indicate ferroelectric transitions. Activation energies become larger in the ferroelectric phase than in the paraelectric phase. Above 400 °C (and below 600 °C), the activation energies are 0.66, 0.70 and 0.95 eV for Nb doping amounts of 0, 1 and 3 at. %. Below 400 °C, the activation energies are 0.72, 0.93 and 1.13 eV, for Nb doping amounts of 0, 1 and 3 at. %. This may be considered general behavior for ferroelectric semiconductors [30]. Above 600 °C, the conductivity rapidly increases again with the activation energy values of 0.87, 0.98 and 1.18 eV for Nb doping amounts of 0, 1 and 3 at. %. Around 200 °C, Nb doping is shown to decrease the conductivity by one to two orders of magnitude. The conductivity of all compositions is shown to be of comparable magnitude at high temperatures above 700 °C. At this high temperature range, the dielectric curves in Figure 4 are shown to be rapidly decreasing due to the wire inductance.

**Table 2:** Electrical properties of (K_0.5_Bi_0.5_)Ti_1−*x*_Nb*_x_*O_3_-0.6 BiFe_1−*x*_Nb*_x_*O_3_ ceramics.

Sample	ε*_rmax_* (10 kHz/1 MHz)	*T_max_* (10 kHz/1 MHz)	tanδ (10 kHz/1 MHz, 40 °C)	σ (Ω^−1^·cm^−1^/300 °C)	σ (Ω^−1^·cm^−1^/30 °C)	*d*_33_ (pC/N)	*S*_max_/*E*_max_ (pm/V)
0.4(K_0.5_Bi_0.5_)TiO_3_-0.6BiFeO_3_	8460	384 °C	0.08	3.38 × 10^−5^	1.57 × 10^−10^	20	90
	6637	419 °C	0.11				
0.4(K_0.5_Bi_0.5_)Ti_0.99_Nb_0.01_O_3_-0.6BiFe_0.99_Nb_0.01_O_3_	7527	387 °C	0.07	1.36 × 10^−5^	1.25 × 10^−12^	13	65
	6034	413 °C	0.13				
0.4(K_0.5_Bi_0.5_)Ti_0.97_Nb_0.03_O_3_-0.6BiFe_0.97_Nb_0.03_O_3_	4050	412 °C	0.07	1.09 × 10^−6^	2.69 × 10^−15^	11	56
	3104	398 °C	0.18				

Several causes have been proposed for the high conductivity of BiFeO_3_ and the solid solutions of BiFeO_3_ such as KBT-BFO. Many workers have proposed that BiFeO_3_ is an n-type semiconductor and that conductivity is governed by the presence of Fe^2+^ and oxygen vacancies [19,31,32,33]. The presence of oxygen-deficient secondary phases has also been suggested as a cause for the high conductivity [34]. Recent density functional theory studies, however, suggest that BiFeO_3_ is a p-type semiconductor [35,36]. Under oxygen rich sintering conditions, Bi and Fe vacancies form preferably over oxygen vacancies. The cation vacancies form shallow acceptor defects. Vengalis *et al.* found that BiFeO_3_ thin films prepared by RF magnetron sputtering displayed p-type semiconducting behavior [37]. Masó and West found that Ca-doped BiFeO_3_ samples that had been heat-treated in O_2_ at ~125 bar were p-type semiconducting [38]. Makhdoom *et al.* found that BiFeO_3_ was a p-type semiconductor up to ~100 °C, after which it became n-type semiconducting [39]. Both groups attributed the conductivity behavior to the presence of Fe^4+^ ions. On the basis of conductivity measurements carried out in oxidizing and reducing atmospheres, it was suggested that the conductivity in KBT-BFO ceramics with high BiFeO_3_ content (≥40 mol %) is p-type [40].

A comparison of the different ionic radii of the metal cations in 6-fold co-ordination shows that Nb will likely substitute for Fe or Ti (Nb^5+^ r_6_ = 0.064 nm, Fe^3+^ r_6_ = 0.065 nm, Ti^4+^ r_6_ = 0.061 nm, K^+^ r_6_ = 0.138 nm, Bi^3+^ r_6_ = 0.103 nm) [41]. The slight increase in the unit cell parameter suggests that Nb prefers to substitute for Ti (Table 1). In either case, Nb_2_O_5_ can enter the KBT-BFO crystal lattice as a donor dopant according to the following defect equations:

(1) $${\text{Nb}}_{2}{\text{O}}_{5}\overset{{\text{BiFeO}}_{3}}{\to }{2\text{Nb}}_{\text{Fe}}^{••}+{3\text{O}}_{\text{O}}^{\text{x}}+↑{\text{O}}_{2}\text{(g)}+4{\text{e}}^{\prime }$$

(2) $${\text{Nb}}_{2}{\text{O}}_{5}\overset{({\text{K}}_{0.5}{\text{Bi}}_{0.5}){\text{TiO}}_{3}}{\to }{2\text{Nb}}_{\text{Ti}}^{•}+{4\text{O}}_{\text{O}}^{\text{x}}+↑\frac{\text{1}}{2}{\text{O}}_{2}\text{(g)}+2{\text{e}}^{\prime }$$

Therefore, p-type conductivity in KBT-BFO is expected to decrease. The activation energy values of dc conductivity may be related to the electronic structure modified by Nb doping as well as by the ferroelectric transition.

**Figure 7:**
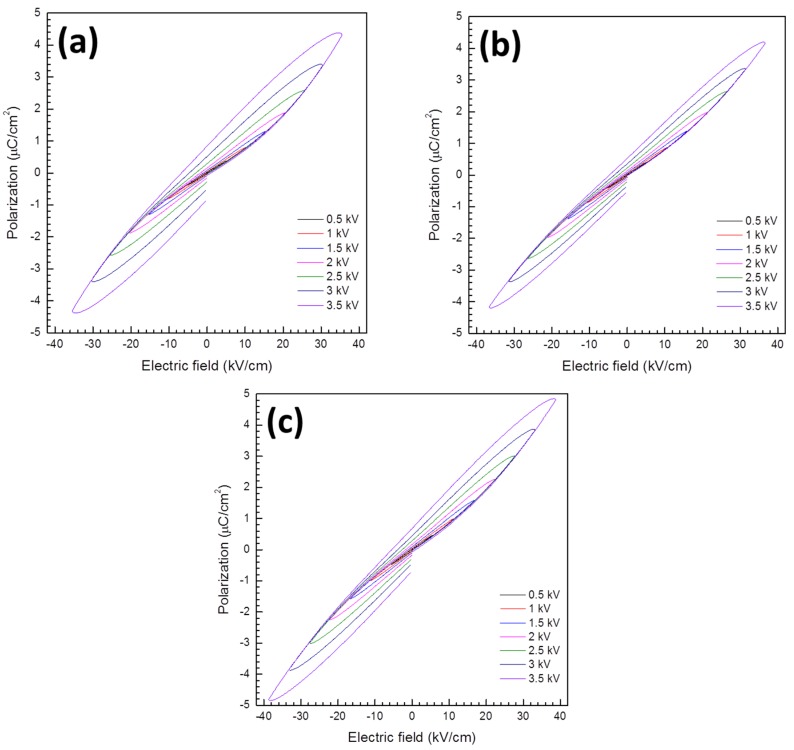
Polarization *vs.* electric field for 0.4(K_0.5_Bi_0.5_)Ti_(1−*x*)_Nb*_x_*O_3_-0.6BiFe_(1−*x*)_Nb*_x_*O_3_ samples sintered at 1050 °C for 1 h: (**a**) *x* = 0.00; (**b**) *x* = 0.01; and (**c**) *x* = 0.03.

**Figure 8:**
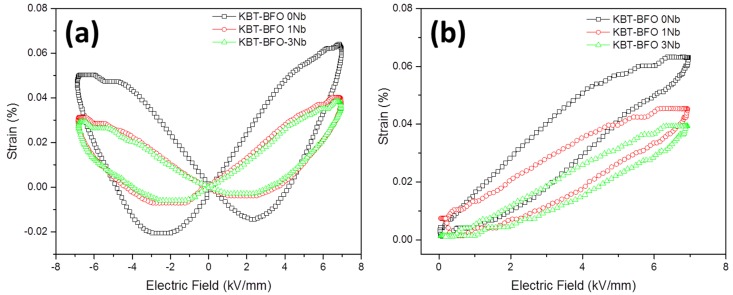
(**a**) Bipolar and (**b**) unipolar strain *vs.* electric field for 0.4(K_0.5_Bi_0.5_)Ti_(1−*x*)_Nb*_x_*O_3_-0.6BiFe_(1−*x*)_Nb*_x_*O_3_ samples sintered at 1050 °C for 1 h.

Curves of polarization *vs.* electric field are shown in Figure 7. All of the samples have narrow, unsaturated P-E loops reminiscent of a linear dielectric material. The gap between the beginning and end of each loop suggests that the samples are conducting [42]. The KBT-BFO 1Nb and 3Nb samples have slightly narrower loops than the KBT-BFO 0Nb sample. Bipolar and unipolar strain *vs.* electric field curves are given in Figure 8. The samples show bipolar butterfly loops, which are characteristic of a classical ferroelectric material, having a negative strain and hysteresis [42,43]. However, the negative strain is low, which indicates that a relaxor phase may make a contribution to the strain [43,44]. Incorporation of Nb causes a decrease in the maximum strain attained in both bipolar and unipolar loops, as well as a reduced negative strain. Values of *S*_max_/*E*_max_ taken from the unipolar loops are given in Table 2. Incorporation of Nb causes a steady decrease in the value of *S*_max_/*E*_max_. The unipolar loop for the KBT-BFO 3Nb sample is noticeably narrower than those of the KBT-BFO 0 and 1Nb samples. Values of *d_33_* measured by *d_33_/d_31_* meter are also given in Table 2. The value of *d_33_* also decreases with increasing Nb content. Due to the low values of *d*_33_, it was not possible to measure *k_t_* and *k_p_* by impedance spectroscopy.

Values of low-field *d_33_* are smaller than those measured by other workers [45,46]. The poling conditions used in this study may need optimization. The reduction in *d*_33_, *S*_max_/*E*_max_ and the strain-electric field hysteresis caused by Nb incorporation may be due to a reduction in the domain wall mobility, and hence extrinsic contributions to the strain [42]. The small reduction in the rhombohedral distortion of the unit cell may also have an effect (Table 1). Nb incorporation may also cause the material to become more relaxor-like, which would also explain the reduced hysteresis and negative strain in the unipolar and bipolar strain-electric field loops (Figure 8) [42,45].

Nb doping effects are qualitatively similar to the effects of Mn additive in our previous work [21]. In the previous work, MnO doping reduced the conductivity of KBT-BFO, leading to a corresponding reduction in ε*_r_* and tanδ. The present work as well as the previous work suggests a correlation between the loss tangent (at low field) and the piezoelectric response in KBT-BFO. The values of relative permittivity near room temperature do not exhibit significant differences with doping (Figure 4). Low magnitude of the loss tangent values at low frequency leads to reduced piezoelectric performance. A similar correlation between piezoelectric tanδ (the ratio between real and imaginary components of *d*_33_) and *d*_33_ was also found for BiFeO_3_ at low frequencies (<1 Hz) [5,8]. Rojac *et al.* found a strong extrinsic contribution to the piezoelectric properties in BiFeO_3_ [5,8]. They postulated a coupling between domain wall motion and electrical conductivity. Mobile charge carriers accumulate at the domain walls and so domain wall motion requires the movement of charges, *i.e.*, electrical conduction. The reduction in conductivity caused by Nb doping of KBT-BFO may therefore lead to the reduction in extrinsic contribution to the piezoelectric properties (Figure 8 and Table 2).

## 3. Experimental Section

Powders of composition 0.4(K_0.5_Bi_0.5_)Ti_(1−*x*)_Nb*_x_*O_3_-0.6BiFe_(1−*x*)_Nb*_x_*O_3_ with *x* = 0.00, 0.01 and 0.03 (KBT-BFO 0, 1 and 3Nb, respectively ) were prepared by ball milling appropriate amounts of K_2_CO_3_ (99%, Alfa Aesar, Haverhill, MA, United States), Bi_2_O_3_ (99.9%, Alfa Aesar), TiO_2_ (99.8%, Alfa Aesar), Fe_2_O_3_ (99.9%, Kojundo, Sakado, Japan) and Nb_2_O_5_ (99.9%, CepaKorea, Daejon, Korea) for 24 h in high purity ethanol (99.9%) in a polypropylene jar with ZrO_2_ milling media. Before milling, all starting materials were dried at 250 °C for 5 h to remove any adsorbed moisture. After milling, the ethanol was evaporated using a hotplate/magnetic stirrer. The dried powder was crushed in an agate mortar and pestle and sieved to pass 180 μm mesh. Powders were calcined in a high purity alumina crucible with lid at 900 °C for 5 h with heating and cooling rates of 5 °C·min^−1^. Calcined powders were examined by X-ray diffraction (XRD, X’Pert PRO, PANalytical, Almelo, The Netherlands) using Cu Kα radiation, a scan range of 10°–90° in 2θ, a step size of 0.026° and a scan speed of 3°·min^−1^.

To make samples for sintering, 0.5 g of powder was pressed by hand in a 10 mm diameter steel die into pellets. The pressed pellets were then cold isostatically pressed at 1500 kg/cm^2^ (~147 MPa). Samples were buried in 0.4(K_0.5_Bi_0.5_)TiO_3_-0.6BiFeO_3_ packing powder in double high-purity alumina crucibles with lids and sintered at 1050 °C for 1 h. Heating and cooling rates were 5 °C·min^−1^.

The density of the sintered samples was measured using the Archimedes method in deionized water. XRD was used to analyze the phase composition of sintered samples as before. For calculation of the unit cell parameters, Si (99.9% Alfa Aesar) was added as an internal standard and the scans repeated with a scan range of 20°–80° in 2θ, a step size of 0.013° and speed of 1°·min^−1^. Unit cell parameters were calculated by the least-squares method using the program Jade 6.5 (Materials Data Inc., Livemore, CA, USA). To observe the microstructure, samples were vertically sectioned using a low speed diamond saw and polished to a 1 μm finish. Samples were thermally etched and the microstructure observed in a scanning electron microscope (SEM, Hitachi S-4700, Hitachi High-Tech, Tokyo, Japan) equipped with an energy dispersive X-ray spectrometer (EDS, EMAX energy EX-200, Horiba, Kyoto, Japan). The mean and standard deviation of the grain size was measured from the micrographs using an image analysis program (ImageJ v1.46r). For each sample, at least 250 grains were measured.

For the measurement of dielectric properties and polarization-electric field hysteresis loops, the samples were parallel polished on both sides and then silver paste was applied and fired onto both sides of the samples. Variation in relative permittivity with temperature was measured using an impedance analyzer (HP4284A, Agilent, Santa Clara, CA, USA) during the cooling of the samples at 1 °C·min^−1^ in oxygen in the temperature range 800–30 °C. P-E loops were measured at room temperature at a frequency of 10 Hz using a Sawyer Tower circuit (RT66B combined with 4 KV HVI, Radiant Technologies Inc., Albuquerque, NM, USA). For the measurement of piezoelectric properties, samples were poled in silicone oil at room temperature under an electric field of 5 kV/mm. The piezoelectric charge coefficient *d_33_* was measured using a Piezo *d*_33_/*d*_31_ meter (Model ZJ-6B, Chinese Academy of Sciences, Beijing, China). Strain *vs.* electric field curves were measured using a contact type displacement sensor (Model 1240; Mahr GmbH, Gottingen, Germany) at 50 mHz.

## 4. Conclusions

The effect of Nb content on the structure, microstructure, and electrical properties of 0.4(K_0.5_Bi_0.5_)Ti_1−*x*_Nb*_x_*O_3_-0.6 BiFe_1−*x*_Nb*_x_*O_3_ (*x* = 0, 0.01 and 0.03) lead-free piezoelectric ceramics has been studied. The incorporation of Nb causes an increase in the second phase content of both calcined powders and sintered ceramics. Nb doping causes a small increase in the unit cell parameter and a small decrease in the rhombohedral distortion of the unit cell. The samples show relaxor-like ferroelectric behavior. However, this is attributed to the high p-type conductivity originating from BiFeO_3_. Nb incorporation causes an increase in activation energy from 0.72 to 0.93 and 1.13 eV below 300 °C and a reduction in conductivity by one to two orders of magnitude at 200 °C. Maximum ε*_r_* is reduced by half. While the frequency dispersion in ε*_r_* is substantially reduced with Nb doping, a strong dispersion in tanδ characteristic of ferroelectricity can be observed. Nb incorporation causes a reduction in the piezoelectric properties and in the strain-electric field hysteresis.
